# Chest wall effect on the monitoring of respiratory mechanics in acute respiratory distress syndrome

**DOI:** 10.5935/0103-507X.20180038

**Published:** 2018

**Authors:** Javier Hernán Dorado, Matías Accoce, Gustavo Plotnikow

**Affiliations:** 1 Capítulo de Kinesiología Intensivista, Sociedad Argentina de Terapia Intensiva - Buenos Aires, Argentina.; 2 Sanatorio Anchorena - Buenos Aires, Argentina.; 3 Hospital General de Agudos Carlos G. Durand - Buenos Aires, Argentina.; 4 Hospital de Quemados - Buenos Aires, Argentina.; 5 Sanatorio Anchorena San Martín - Buenos Aires, Argentina.

**Keywords:** Thoracic wall, Respiration, Artificial, Respiratory distress syndrome, Adult, Respiratory mechanics, Ventilator-induced lung injury

## Abstract

The respiratory system mechanics depend on the characteristics of the lung and chest wall and their interaction. In patients with acute respiratory distress syndrome under mechanical ventilation, the monitoring of airway plateau pressure is fundamental given its prognostic value and its capacity to assess pulmonary stress. However, its validity can be affected by changes in mechanical characteristics of the chest wall, and it provides no data to correctly titrate positive end-expiratory pressure by restoring lung volume. The chest wall effect on respiratory mechanics in acute respiratory distress syndrome has not been completely described, and it has likely been overestimated, which may lead to erroneous decision making. The load imposed by the chest wall is negligible when the respiratory system is insufflated with positive end-expiratory pressure. Under dynamic conditions, moving this structure demands a pressure change whose magnitude is related to its mechanical characteristics, and this load remains constant regardless of the volume from which it is insufflated. Thus, changes in airway pressure reflect changes in the lung mechanical conditions. Advanced monitoring could be reserved for patients with increased intra-abdominal pressure in whom a protective mechanical ventilation strategy cannot be implemented. The estimates of alveolar recruitment based on respiratory system mechanics could reflect differences in chest wall response to insufflation and not actual alveolar recruitment.

## INTRODUCTION

The respiratory system mechanics depend on the characteristics of the lung and chest wall and on their interaction.^(^^1^^)^

Mechanical ventilation (MV) is implemented in patients with acute respiratory distress syndrome (ARDS) for life support. Tidal volumes of 6 mL/kg predicted body weight and airway plateau pressure under 30 cmH_2_O are strategies for minimizing ventilator-induced lung injury (VILI) and have shown to improve survival.^(^^2^^)^ However, more than a decade after the ARMA trial,^(^^2^^)^ mortality remains at very high percentages (approximately 40%).^(^^3^^)^

Except for the H1N1 virus epidemic, wherein ARDS mortality was related to refractory hypoxemia,^(^^4^^)^ multiple organ failure is the main cause of death, and VILI caused by inadequate ventilation setting could contribute to its development.^(^^5^^)^

A retrospective analysis found that a driving pressure (DP) higher than 15cmH_2_O in patients with ARDS is associated with increased mortality and could be related to the functional size of the lung and to the potentially harmful character of MV, which we consider "protective".^(^^6^^)^

The airway pressure measured in patients without ventilatory effort reflects the impedance of the respiratory system as a whole. Knowing each of its isolated components requires an esophageal balloon.^(^^7^^)^ However, a recent study reported that esophageal pressure (Pes) is used as a measurement tool only in 1.2% patients, even in patients with severe ARDS.^(^^8^^)^

Obese patients and those with pleural effusion or intra-abdominal hypertension (conditions in which the chest wall mechanics could be affected) under MV for ARDS are a challenge.^(^^9^^)^ It is usually tolerated plateau pressure levels above those recommended based on the physiological rationale of providing a "protective effect" to the stiffness of the chest wall by reducing transpulmonary pressure (P_L_), the actual pressure that acts on the lung.^(^^1^^)^ However, chest wall behavior has not been completely elucidated and may lead to (in the case of erroneous interpretations) high levels of energy applied to the lung parenchyma and, consequently, to VILI.

Knowing the chest wall effect on the respiratory system of patients with ARDS could make it possible to maximize the data collected through basic ventilatory monitoring and to differentiate patients in whom the ventilatory strategy can be guided by assessing the airway plateau pressure from those in whom esophageal manometry is required to optimize the MV settings.

The aim of the present narrative review is to describe the behavior of the chest wall, its effect on ventilatory monitoring and its role in the selection of protective MV strategies in patients with ARDS without ventilatory effort.

## STATE OF THE ART

### Is normal chest wall behavior elastic?

The chest wall has been defined as all body segments that share and affect changes in lung volume.^(^^10^^)^ A traditional perspective describes the respiratory system as an elastic structure (lung) within another elastic structure (chest wall).^(^^9^^-^^14^^)^

**Figure 1:**
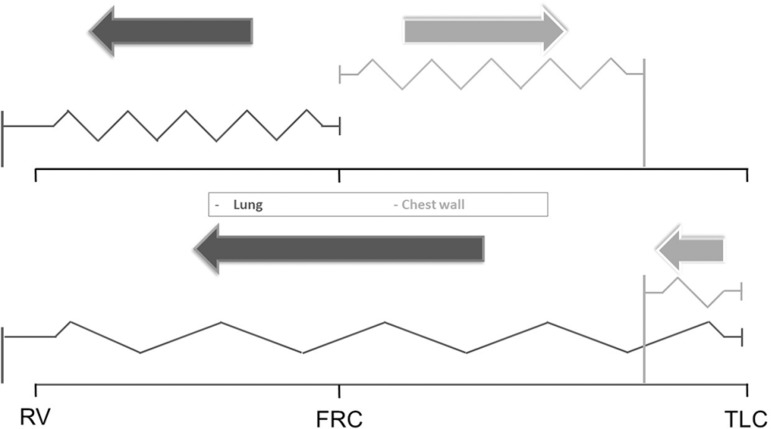
Graphical representation of the traditional "elastic" lung and chest wall model. The vertical lines anchored to the base represent the resting volumes of each structure and the arrows the elastic recoil pressure according to the volume of the respiratory system. RV - residual volume; FRC - functional residual capacity; TLC - total lung capacity.

As all "elastic" structures, the lung and the chest wall have a resting volume. If the lung were isolated from the action of the chest wall, it would stabilize in a situation of collapse. Conversely, the relaxation volume of the chest wall is at 75% of vital capacity.^(^^15^^,^^16^^)^ The elastic recoil of the lung in any situation will generate a positive elastic recoil (that is, tendency of the lungs to recoil inwards); however, the chest wall may exert negative (that is, the tendency of the chest wall to pull outwards) or positive elastic recoil, according to the relationship between a given volume and its resting volume (Figure 1).

Considering this behavior valid, several questions emerge:

- If the chest wall relaxation volume is higher than the functional residual capacity (FRC), then what circumstances make it possible for the chest wall to cause positive pleural pressure, to compress the lung and, consequently, to increase the airway plateau pressure?

Dorsal decubitus, use of sedatives, neuromuscular blockers, obesity and/or increased intra-abdominal pressure (IAP) substantially reduce the chest wall and respiratory system resting volumes, resulting from the decrease in the negative elastic recoil of the chest wall concurrent to the decrease in FRC. Therefore, the resting volume of the chest wall remains higher than that of the respiratory system. Consequently, chest wall exerts positive pleural pressure only when the volume of the respiratory system exceeds the chest wall relaxation volume. This situation is described in patients with chronic obstructive pulmonary disease, but it is unlikely to occur in patients with ARDS. Conversely, studies have shown that in parenchymal conditions that lead to increased lung weight (pneumonia or ARDS), the natural tendency of the lung to collapse is magnified.^(^^17^^)^ Consequently, if pulmonary collapse is not present, then the chest wall is likely responsible for keeping it insufflated rather than limiting its expansion.^(^^18^^)^

Another possible explanation is based on the potential error of assuming Pes as a surrogate for pleural pressure. The latter shows a heterogeneous response to the impact of gravity force, and esophageal manometry is only able to estimate it when horizontal to it.^(^^19^^)^ Thus, the question remains of whether positive Pes in lung-dependent areas responds to the chest wall effect or reflects the pressure of the lung to the chest wall fixed against the support plane.

- Assuming an elastic behavior, changes in the volume of the respiratory system should generate predictable changes in Pes as long as the chest wall elastance is known.

A group of Swedish authors addressed this point indirectly, considering that the chest wall does not act as an elastic object.^(^^20^^,^^21^^)^ The aforementioned hypothesis is supported by the following findings:

- Significant differences were observed when comparing the end-expiratory Pes change assessed by esophageal manometry and predicted by multiplying the chest wall elastance (E_CW_) by the end-expiratory lung volume (EELV) change after a positive end-expiratory pressure (PEEP) step. In all cases, the end-expiratory Pes was markedly lower than expected for an elastic behavior.^(^^20^^,^^21^^)^
- An elastic structure, at the same volume, exerts a specific recoil pressure, regardless of the way in which it was insufflated. Figure 2 shows that when the chest wall is insufflated by tidal volume, the generated displacement pressure is substantially higher than that required by PEEP steps.^(^^21^^)^
  **Figure 2:**
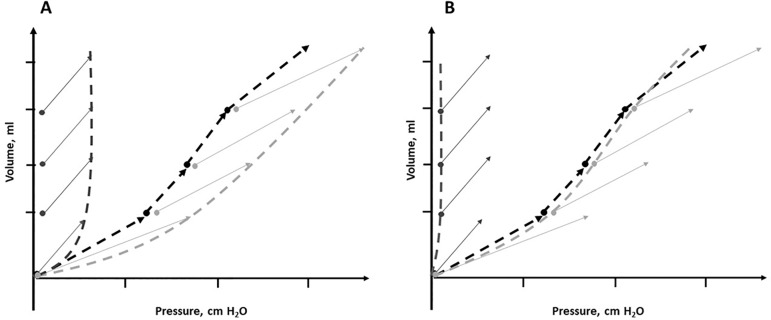
Respiratory system (in black), transpulmonary (in light gray) and chest wall (in dark gray) pressure volume curves constructed using end-inspiratory pauses (diagram A) and positive end-expiratory pressure steps and end-expiratory pauses (diagram B). The effect of chest wall mechanics is non-significant, as shown by overlapping transpulmonary and end-expiratory airway curves (diagram B).
- In an elastic model, the volume gain by pressure change should respond to the mechanical characteristics of both composing structures. However, the change in EELV between 4 and 16 cmH_2_O PEEP only obtained a good correlation (r^2^ = 0.83) with the change predicted by the equation [PEEP change (∆PEEP) × lung elastance (E_L_)], indicating that the chest wall effect is negligible when the respiratory system is insufflated with PEEP^(^^21^^)^ (Figure 2).

Thus far, no model based on the chest wall as an elastic structure has been able to explain the above findings. An alternative to the traditional behavior in which the chest wall is functionally divided into two components has been recently proposed: the rib cage, which generates a force that opposes the elastic recoil of the lung, and the abdomen, which is mechanically considered a hydraulic structure.^(^^14^^,^^22^^)^ Systems consisting of hydraulic and elastic structures are governed by the principle of viscoelasticity. The response to the load of viscoelastic tissues is affected not only by the magnitude of the force applied but also by the temperature (could be considered constant in the case of the respiratory system) and by the rate of application of the load. At high application rates (insufflation with tidal volume - V_T_), the structure responds with greater stiffness, requiring a higher pressure for a given volume (response similar to that of an elastic structure); conversely, when applied slowly (insufflation with PEEP), the resistance to deformation decreases (Table 1).

**Table 1:** Differences in mechanical responses to the deformation of elastic and viscoelastic structures

	Elastic behavior	Viscoelastic behavior
Rapid deformation (tidal volume)	Linear response, volume changes as a function of respiratory system compliance	Linear response, volume changes as a function of respiratory system compliance
Slow deformation (PEEP)	Linear response, volume changes as a function of the respiratory system compliance	Bimodal response 1^st^ phase, volume changes as a function of respiratory system compliance 2^nd^ phase, volume changes as a function of lung compliance
Volume gain	Predictable ∆Vol = ∆P x Crs	Unpredictable Temperature (≈ constant) ∆P Application rate

PEEP - positive end-expiratory pressure; ∆Vol - volume change; ∆P - pressure change; Crs - respiratory system compliance.

The theoretical model can explain the behavior of the system during insufflation and deflation in MV; however, understanding why the respiratory system has a "viscoelastic" response is crucial for monitoring clinical variables. The incorporation of volume into the respiratory system moves the rib cage outward and the diaphragm downward (70% and 30%, respectively). The rib cage nears its relaxation volume, which could explain the negligible change in end-expiratory Pes.^(^^14^^,^^18^^,^^20^^,^^23^^)^ The diaphragm and its close relationship with the abdominal cavity could be responsible for the different response to the insufflation mode.

**Figure 3:**
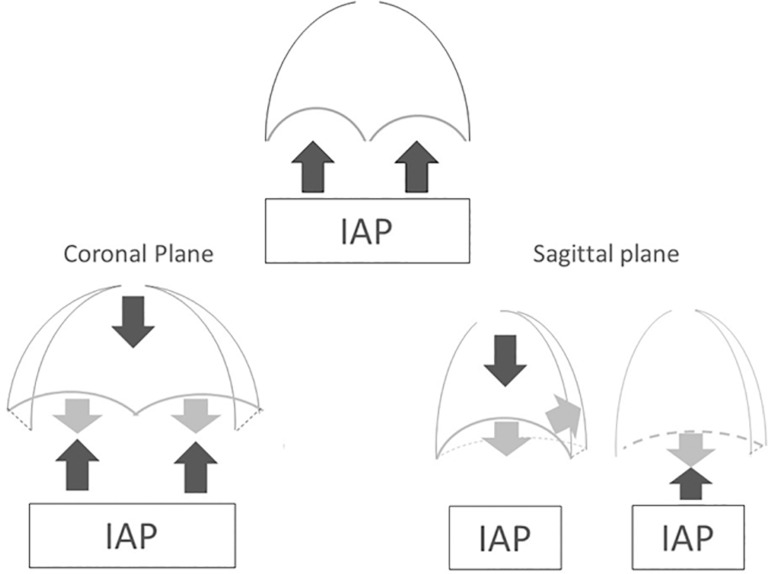
Diaphragm net effect: during insufflation, the increase in transversal and coronal axes in the caudal area of the rib cage passively tensions the diaphragm, preventing the intra-abdominal pressure from affecting the thoracic cavity. IAP - intra-abdominal pressure.

**Figure 4:**
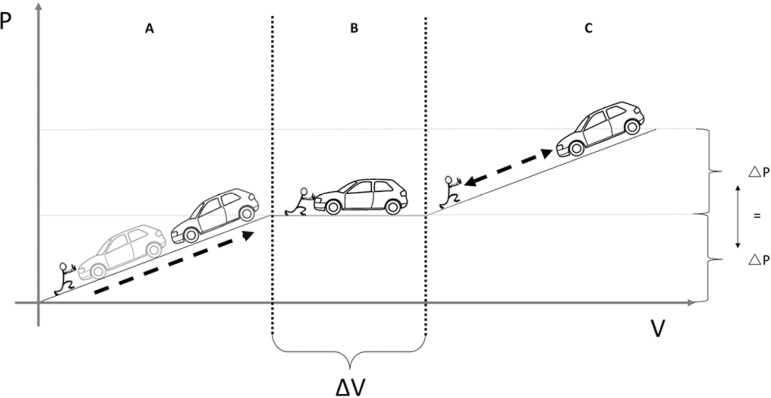
Exemplification of different chest wall responses to deformation as the load a subject must overcome to push a car up an inclined plane. A) Viscoelastic response to rapid deformation: The force [in the respiratory system, pressure change ∆P] required to push the car up (in the respiratory system, volume gain, ∆V) is related to the weight of the vehicle and to the slope of the inclined plane (in the respiratory system, chest wall elastance). Once the force is removed, the vehicle will return to its initial position with a magnitude of force identical to that necessary to push the car up. B) Viscoelastic response to slow deformation. If, after the ascent, the load (pressure) is sustained over time (PEEP insufflation), the car continues moving through a plateau (ΔV), where the required force is negligible. C) If the car is pushed up a new slope with the same characteristics (no change in chest wall elastance), the necessary force will be of equal magnitude to that of phase A.

The position of the diaphragm depends on the interplay between the force in the direction of expansion of the rib cage, the elastic recoil of the lung and the IAP.^(^^21^^)^ During inspiration, the descent of the diaphragm displaces the abdominal contents caudally. To achieve this displacement, the resistance exerted (related to the IAP) must be overcome, and therefore, the end-expiratory Pes will increase, which becomes evident only when insufflation occurs at the expense of the tidal volume or during the first ventilatory cycles after a PEEP step ("viscoelastic" response to rapid deformation).^(^^14^^)^ After a period of stabilization, the end-expiratory Pes returns to baseline values, while the volume in the respiratory system continues to increase.^(^^14^^)^ This finding could be explained by the theory of the "net effect" of the diaphragm, wherein the expansion of the caudal area of the rib cage puts tension in its circumferential fibers (passive tension), thereby preventing the IAP from exerting its effect on the thoracic cavity^(^^21^^)^ (Figure 3). Consequently, the dynamic load imposed by the abdomen could be considered constant, regardless of its initial EELV and, once a new static equilibrium is reached, its effect becomes negligible ("viscoelastic" response to slow deformation).^(^^14^^)^ This behavior can be exemplified by the load an individual must overcome to push a car up an inclined plane. Disregarding the friction with the surface, the force (in the respiratory system, pressure) that must be made for the displacement (in the respiratory system, volume gain) is related to the weight of the vehicle and to the slope of the inclined plane (variables representing the E_CW_). Once this force is removed, the vehicle will return to its initial position with a magnitude of force identical to that necessary to produce the ascent (Figure 4A, viscoelastic response to rapid deformation). If, after pushing the car up the slope, the load is sustained over time (insufflation with PEEP), the response changes abruptly; that is, the car continues moving along the plateau, wherein the force required is negligible (Figure 4B, viscoelastic response to slow deformation). Lastly, if the individual tries to push the car up over a new incline in this new situation (Figure 4C), the necessary force will again depend on the weight of the vehicle and on the slope of the incline (E_CW_), which, as mentioned above, remains constant, with a magnitude equal to that of phase 1.

In summary, the concept of the chest wall as an elastic structure cannot explain the behavior reported in the literature, whereas a "viscoelastic" behavior more closely fits the findings.

### Chest wall in acute respiratory distress syndrome

In severely affected patients, the protective MV strategy may cause injury.^(^^6^^)^ VILI responds to two mechanisms: stress (tension) and strain (deformation).^(^^24^^)^ The two variables can be calculated using the following equations:

- Stress (P_L_): Alveolar plateau pressure - End-inspiratory Pes.
- Strain: V_T_/FRC.

Acute respiratory distress syndrome is characterized by the decrease in respiratory system compliance, affecting the lung component to a greater or lesser extent depending on the etiology. Conversely, obesity, pleural effusion and abdominal hypertension could deteriorate the chest wall mechanics and, therefore, the validity of assessing the airway plateau pressure to predict pulmonary stress.^(^^9^^)^

Pleural effusion increases the imposed pressure, causing the passive collapse of the adjacent pulmonary parenchyma. In a model with healthy pigs, Graf et al. observed that using moderate PEEP levels is sufficient to significantly reduce the lung collapse and that under these conditions, the chest wall expansion contain the entire volume of the pleural effusion.^(^^25^^)^ In 2013, Chiumello et al. included 129 patients with ARDS and pleural effusion in their study. The patients with a higher volume of pleural effusion showed no significant differences in the elastance of the respiratory system (E_RS_), lung (E_L_) and chest wall (E_CW_) from patients with a lower volume of pleural effusion. The lower chest wall elastance, in comparison with the lung, likely helps the pressure exerted by the pleural effusion to move the chest wall closer to its relaxation volume without affecting its mechanical properties (Table 2).^(^^26^^)^

**Table 2:** Description of the potential effects of comorbidities on chest wall responses in acute respiratory distress syndrome

	Pleural effusion	Obesity	Intra-abdominal hypertension
Pathophysiological rationale	⬆ Imposed pressure ⬇ Lung volume ⬆ CW elastance	⬇ Lung volume CW infiltration ⬆ CW elastance	⬇ Lung volume ⬆ CW elastance
Bibliographic findings	Moderate PEEP levels reverse lung collapse^(^^25^^)^ Normal CW elastance^(^^26^^)^	⬇ Lung volume due to diaphragmatic elevation^(^^29^^)^ Normal CW elastance^(^^29^^)^	⬇ Lung volume with increased IAP^(^^31^^)^ Increased CW elastance^(^^31^^)^
MV considerations	Moderate PEEP levels Guide the MV by the airway plateau pressure	Selection of decremental PEEP according to the RS elastance Guide the MV by the airway plateau pressure	PEEP selection to counteract the effect of the IAP Guide MV by the esophageal pressure

CW - chest wall; PEEP - positive end-expiratory pressure; IAP - intra-abdominal pressure; MV - mechanical ventilation; RS - respiratory system.

Regarding obesity, although no direct relationship between body mass index and E_CW_ is observed in normal subjects,^(^^27^^)^ monitoring the Pes when choosing V_T_ and PEEP in obese patients with ARDS could provide valuable information to minimize VILI for two reasons:

1. Quantifying the end-inspiratory P_L_, a measure of stress, given the potential protective effect of the increase in E_CW_.
2. Calculating the end-expiratory P_L_, which, when negative, indicates pulmonary collapse, with the consequent opening and collapse in each ventilatory cycle.^(^^11^^,^^28^^)^

Chiumello et al. assessed respiratory mechanics variables in patients with ARDS stratified according to body mass index. Even at different PEEP levels (5 and 15cmH_2_O), the E_CW_ of obese patients had a median of 5cmH_2_O/liter, within the normal range.^(^^9^^,^^29^^)^ Conversely, tomographic analysis showed that overweight and obese patients had a lower EELV than patients with normal weight. The authors attributed this finding to the lower vertex-base pulmonary distance (determined by the cephalic displacement of the diaphragm).^(^^29^^)^ Hence, in obese patients with ARDS, the chest wall behavior supports the PEEP role in reestablishing the EELV.

Pirrone et al. demonstrated that after a recruitment maneuver, the PEEP decrement titration strategy according to the best E_RS_ is as effective as positive end-expiratory P_L_ objetive titration in morbidly obese patients without ARDS.^(^^30^^)^ After selecting the adequate PEEP level, the normal E_CW_ suggests that the airway pressure could indicate pulmonary stress with a level of precision similar to that observed in the general population (Table 2).

**Figure 5:**
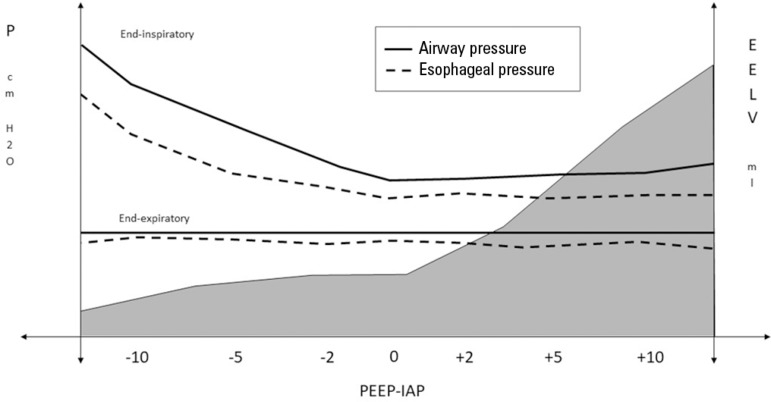
Graphical representation of the end-expiratory lung volume (EELV), airway pressure and esophageal pressure as a function of the PEEP-IAP gradient. Note the marked decrease in end-expiratory lung volume (EELV) when the IAP level exceeds the programmed PEEP. The increase in airway pressure when the IAP exceeds the absolute value of PEEP may be explained by the increase in end-inspiratory esophageal pressure; however, the end-expiratory Pes remains non-responsive to the increase in IAP. EELV - end-expiratory lung volume; PEEP - positive end-expiratory pressure; IAP - intra-abdominal pressure.

Another comorbidity that could affect the chest wall behavior in ARDS is the increase in IAP.^(^^31^^-^^33^^)^ This condition causes a marked deterioration in both lung volume and respiratory system mechanics. The magnitude of such an effect depends on the relationship between the PEEP level and IAP. As long as the IAP remains lower than the PEEP, it will have no impact on EELV or respiratory mechanics. Conversely, when the IAP exceeds the PEEP, the EELV decreases linearly, and the airway pressure and the end-inspiratory Pes increase, thus increasing the E_CW_ and the E_RS_.^(^^31^^,^^34^^)^ However, the end-expiratory Pes remains virtually unresponsive to changes in IAP. Therefore, abdominal hypertension affects the chest wall behavior differently, according to the insufflation method, with a strong effect under dynamic conditions (tidal volume) and with a negligible effect under static conditions (PEEP)^(^^31^^,^^34^^)^ (Figure 5 and Table 2).

The results observed in patients with pleural effusion,^(^^26^^)^ obesity^(^^29^^,^^30^^)^ and intra-abdominal hypertension^(^^31^^-^^34^^)^ adequately fit the model of the "viscoelastic" chest wall behavior. Its mechanical characteristics may not be affected by such conditions, except when the IAP increases, which may be relevant for monitoring patients with ARDS.

### Ventilatory monitoring in acute respiratory distress syndrome and chest wall effect

In patients with ARDS, ventilatory monitoring requires assessing whether MV is protective or injurious.^(^^7^^,^^35^^)^

The airway plateau pressure measurement only requires the technology included in the ventilator. However, such a variable can be affected by different factors, including the insufflation method and the lung and chest wall responses.^(^^32^^)^

For practical purposes, the monitoring tools that allow us to independently estimate the correct tidal volume, on one hand, and PEEP, on the other hand, will be described, as will the potential interpretation error that could lead to the chest wall effect.

## TIDAL VOLUME

The use of plateau pressure may not be a good surrogate for pulmonary stress inferred based on end-inspiratory P_L_ and has been shown to be imprecise in predicting an end-inspiratory P_L_ higher than 25cmH_2_O.^(^^36^^)^ Moreover, the main disadvantage is that it disregards the pressure from wich the V_T_ is delivered, that is, PEEP. In spite of the above limitations, levels higher than 30 cmH_2_O remain useful predictors of mortality.^(^^37^^)^

Airway driving pressure has been proposed as a measure that assesses the functional size of the lung and has been shown to be the main predictor of mortality in a retrospective analysis conducted by Amato et al., regardless of tidal volume over predicted body weight.^(^^6^^)^

When the E_CW_ increases, the same airway driving pressure can generate different P_L_ levels.^(^^38^^)^ Nonetheless, the prediction of changes in P_L_ from the airway driving pressure has shown satisfactory results.^(^^36^^,^^39^^,^^40^^)^ Chiumello et al. observed an acceptable correlation between the two variables (r^2^: 0.737 and r^2^: 0.656, at 5 and 15cmH_2_O PEEP, respectively), determining that an airway driving pressure higher than 15cmH_2_O satisfactorily predicts pulmonary stress above the proposed limits for a protective ventilation with an area under the ROC curve of 0.864 (95% confidence interval: 0.801 - 0.929).^(^^39^^)^ Such a finding corroborates a retrospective analysis in which the airway driving pressure showed a strong linear correlation with the transpulmonary driving pressure.^(^^40^^)^ In turn, in a 24-hour follow-up, the patients who maintained high values of both airway and transpulmonary driving pressure had higher mortality, showing that the decrease in airway driving pressure exclusively responds to the improvement in the mechanical conditions of the lung.^(^^40^^)^ Therefore, in a general population of patients with ARDS, the chest wall effect on the respiratory system mechanics is negligible.^(^^36^^,^^40^^)^

**Figure 6:**
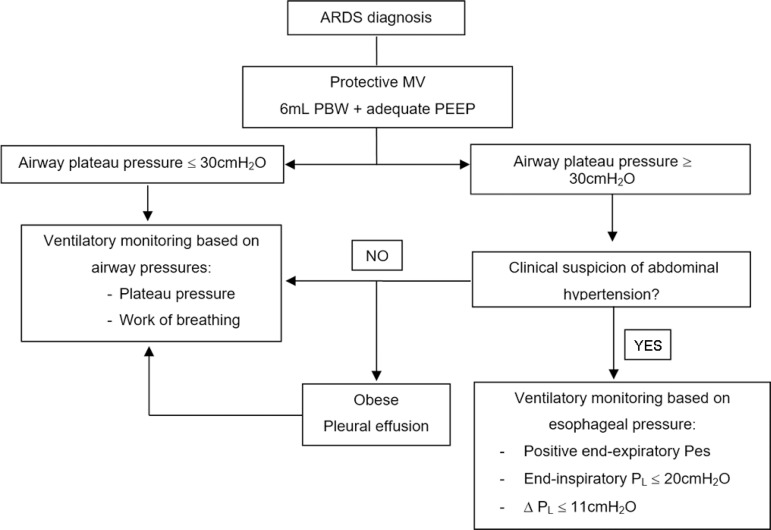
Action algorithm proposed for patients with respiratory distress syndrome. ARDS - acute respiratory distress syndrome; MV - mechanical ventilation; PBW - predicted body weight; PEEP - positive end-expiratory pressure; Pes - esophageal pressure; P_L_ - transpulmonary pressure; ∆P_L_ - inspiratory transpulmonary pressure change.

Lastly, Cortés-Puentes et al. conducted a study with pigs also showing that the airway driving pressure behaves similarly to the transpulmonary driving pressure under normal conditions, unilateral massive atelectasis, and unilateral and bilateral lung injury, also reporting that abdominal hypertension distorts this relationship and that the model compatible with ARDS is the least affected by this variable. This model showed significant differences in absolute values; however, the relationship between airway and transpulmonary driving pressure remains constant when comparing abdominal hypertension with normal IAP.^(^^31^^,^^32^^)^ In summary, esophageal manometry could be useful in patients with abdominal hypertension, when the airway plateau pressure exceeds the safety limits, to more accurately estimate the pulmonary stress (Figure 6).

### Positive end-expiratory pressure

Basic monitoring offers fewer alternatives to assess the appropriate selection of PEEP. Its titration has three main objectives:^(^^33^^)^

- Reestablishing the EELV by recruiting collapsed units.
- Minimizing the opening and cyclic collapse of unstable units.
- Avoiding alveolar overdistension.

In their seminal study, Suter et al. reported that selecting PEEP according to the best oxygenation is far from indicating the best mechanical conditions for the respiratory system.^(^^41^^)^ This finding was corroborated by Rodríguez et al. in patients with ARDS secondary to pneumonia.^(^^36^^)^ Conversely, the titration for the best E_RS_ has been shown to match the maximum oxygen transport, the best E_L_ and the best relationship between dead space ventilation and tidal volume.^(^^36^^,^^41^^)^

The drop in EELV in patients with ARDS under MV may be aggravated when associated with comorbidities such as obesity and abdominal hypertension. Except for the increase in IAP, no condition alters the E_CW_. Therefore, the E_RS_ could adequately reflect the PEEP effects on the pulmonary parenchyma (Figure 6).^(^^11^^,^^12^^,^^29^^)^ However, the main chest wall effect on the selection of PEEP is likely not linked to the E_CW_ but rather to the decrease in EELV, for which basic monitoring lacks useful tools.

In a study conducted to characterize the pulmonary and extrapulmonary mechanical behaviors of patients with ARDS, Gattinoni et al. found that patients with extrapulmonary ARDS had high IAP levels. Therefore, these finding can be used to describe the abdominal hypertension effect on ARDS.^(^^42^^)^ The high E_RS_ observed responds to the increases in E_CW_ and in E_L_. In turn, the gradual increase in PEEP showed significant improvements in the elastance of both structures, even at PEEP levels that did not reach the IAP value.^(^^42^^,^^43^^)^

Several research studies have been conducted towards titrating PEEP to counteract the increase in the IAP.^(^^33^^,^^42^^-^^44^^)^ A PEEP/IAP ratio of 0.5 decreases the deleterious effects on oxygenation and on respiratory mechanics of abdominal hypertension and also limits the cardiac output deterioration.^(^^43^^,^^44^^)^ However, IAP is usually quantified based on the bladder pressure, which could overestimate the abdominal pressure on the thoracic cavity in subjects under MV in a semi-sitting position and consequently guide the selection of excessive PEEP levels.

Another tool that could make it possible to calculate the overload on the lung imposed by the abdominal pressure is Pes. Yang et al. compared patients with ARDS with abdominal hypertension and those without it and observed that subjects with IAP higher than 12 mmHg had higher E_RS_, E_L_, E_CW_ values and lower EELV. PEEP titration by esophageal manometry increased the EELV by 58.7% over the basal levels; conversely, the increase was only 26.4% in patients without abdominal hypertension.^(^^33^^)^

Lastly, the best PEEP is that at which alveolar recruitment prevails. Estimating the alveolar recruitment potential makes it possible to stratify patient severity and to guide therapy.^(^^35^^,^^45^^-^^47^^)^ Although the gold standard for assessing recruitment is tomography, different tools have been proposed based on the mechanical behavior of the respiratory system. Mechanics-based methods have showed very good correlations between each other; however, they are not correlated with tomographic estimation, and therefore, they likely assess different phenomena.^(^^17^^,^^47^^,^^48^^)^

**Figure 7:**
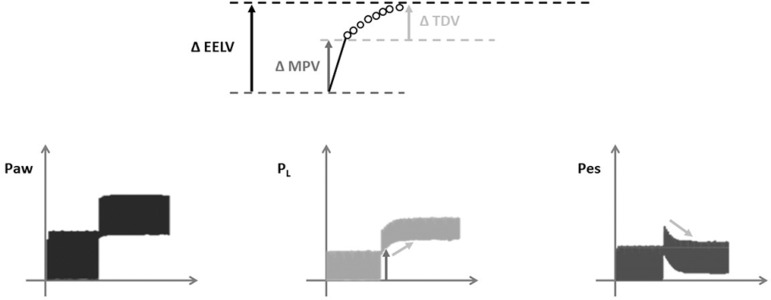
Relationship between the mechanical behavior of the respiratory system (bottom images) and end-expiratory lung volume (EELV) changes (top image) after a PEEP step. The increase in airway pressure (Paw, bottom left) coincides with the increases in both pleural (Ppl, bottom right) and transpulmonary (P_L_, bottom middle) pressures, resulting from the initial volume gain (MPV, minimum predicted volume), reflecting the combined mechanical response of the lung and chest wall. After the first ventilatory cycle at the new PEEP level, the pleural pressure begins to decrease, and consequently, the transpulmonary pressure increases, which generates volume gain (TDV, time-dependent volume, above), which, in this case, depends on the lung mechanical characteristics. PEEP - positive end-expiratory pressure.

The gain in EELV by increasing the PEEP has two phases.^(^^49^^)^ The first is established during the first ventilatory cycle after the PEEP step, termed predicted minimum change,^(^^20^^,^^48^^)^ in which the diaphragm and abdominal contents are moved caudally, increasing the Pes. However, during the successive ventilations, Pes gradually returns to its basal level, whereas the airway pressure remains constant, and P_L_ increases. Therefore, the second phase of insufflation, termed time-dependent volume, is exclusively related to the characteristics of the lung (Figure 7).^(^^48^^)^ Consequently, if the time-dependent volume adjusts to the mechanical characteristics of the functional lung, the response of the chest wall to slow insufflation ("viscoelastic" model), not alveolar recruitment, may explain these findings.

In summary, the chest wall effect is likely overestimated during basic monitoring of patients with ARDS. Its main effect on respiratory system mechanics is the drop in the EELV of patients with abdominal hypertension. In these cases, PEEP should be selected towards reestablishing such volume. For such a purpose, Pes monitoring is available.^(^^18^^)^ After selecting the appropriate PEEP level, airway pressure (despite the above limitations) has been shown to be a surrogate for inspiratory stress. Therefore, in the longitudinal follow-up of patients with ARDS, the change in airway pressure reflects, with an acceptable degree of certainty, changes in the characteristics of the lung. When nearing the safety limits proposed for plateau pressure, Pes monitoring could be useful (Figure 6).

## CONCLUSION

The chest wall effect on respiratory system mechanics is overestimated, which may lead to erroneous decision making. Monitoring airway pressure during mechanical ventilation is crucial given its key prognostic value and its ability to express pulmonary stress. The use of advanced monitoring tools (esophageal pressure) could be reserved for patients with clinically suspected increased intra-abdominal pressure in whom a protective mechanical ventilation strategy cannot be safely implemented. However, the pressure in the airway is not valid for correctly assessing the positive end-expiratory pressure toward restoring the end-expiratory lung volume. In this scenario, the best mechanical condition of the respiratory system likely coincides with the value of positive end-expiratory pressure that counteracts the intra-abdominal pressure effect. Estimates of alveolar recruitment induced by positive end-expiratory pressure based on respiratory system mechanics could reflect differences in the behavior of the chest wall according to the insufflation method and not actual alveolar recruitment.
